# Herlyn-Werner-Wunderlich Syndrome: A Rare Case Report

**DOI:** 10.7759/cureus.35003

**Published:** 2023-02-15

**Authors:** Zainab E Habbash

**Affiliations:** 1 Radiology, Salmaniya Medical Complex, Manama, BHR

**Keywords:** case report, magnetic resonance imaging, acute abdominal pain, severe dysmenorrhea, herlyn-werner-wunderlich syndrome

## Abstract

Müllerian anomalies are a complex spectrum of congenital defects of the female reproductive tract caused by an interruption in the normal development of Müllerian ducts and their associated structures. The clinical presentation of these anomalies varies and includes acute presentations. In this report, a case of a 16-year-old girl who attended the emergency department with severe colicky abdominal pain and nausea has been presented. The patient was on the fifth day of the menstrual cycle, and her pain was not completely relieved by the administration of non-steroidal anti-inflammatory drugs. The patient had menarche at the age of 14 years and used to have a regular menstrual cycle every 30 days. Her menstrual flow was average with a cycle duration of five days. An abdominal ultrasound examination was performed to rule out ovarian torsion, which demonstrated normal appearance and ovary size, and a large heterogeneous collection in the rectouterine pouch with unclear uterine morphology. Subsequently, magnetic resonance imaging revealed a duplication of the uterus, cervix, and vagina. The right hemivagina and right endometrial cavity were distended, consistent with hematometrocolpos. Right renal agenesis with compensatory hypertrophy of the left kidney was observed. The constellation of uterus didelphys obstructed hemivagina, and ipsilateral renal agenesis represented the diagnosis of Herlyn-Werner-Wunderlich syndrome. Herlyn-Werner-Wunderlich syndrome is a rare and complex type of congenital uterine abnormality. Although rare, this syndrome should be considered in the differential diagnosis of severe dysmenorrhea in adolescent girls with renal anomalies. In this case, the patient underwent vaginoplasty with resection of the vaginal septum to relieve the obstruction. At follow-up visits, she did not have a recurrence of symptoms.

## Introduction

Müllerian anomalies are a complex spectrum of congenital defects of the female reproductive tract caused by an interruption in the normal development of Müllerian ducts and their associated structures. The prevalence of Müllerian anomalies is up to 5% in the general population, but it is markedly higher among women with infertility or recurrent pregnancy loss at up to 25% [1]. There is no universally accepted classification system for such anomalies, and most of the current classification systems have certain shortcomings, as the categories have overlapping features since Müllerian anomalies represent a spectrum of developmental anomalies [2]. The clinical presentation of Müllerian anomalies varies depending on the type of defects. In general, Müllerian anomalies can be asymptomatic, present at menarche with cyclic pain, or have adverse pregnancy outcomes [3]. I reported the case of a patient with Herlyn-Werner-Wunderlich syndrome, which is the rarest type of Müllerian anomaly, who presented to the emergency department with acute abdominal pain [3].

## Case presentation

A 16-year-old girl was brought to the emergency department with severe colicky lower abdominal pain and nausea. The patient was on the fifth day of her menstrual cycle, and her pain was not completely relieved with non-steroidal anti-inflammatory drugs. There was no history of fever, vaginal discharge, or change in bowel or urinary habits. The patient attained menarche at the age of 14 years and had a regular menstrual cycle every 30 days. Her menstrual flow was average and had a cycle duration of five days. Notably, the pain decreased throughout the cycle. She had an average menstrual loss associated with severe dysmenorrhea, which affected her school attendance since she attained menarche. The patient did not start her sexual life. The past medical history was non-contributory. The family history was notable for thalassemia in her sister.

On examination, the patient looked ill, but her vital signs were within normal limits. Abdominal examination revealed tenderness and a mass in the suprapubic region. There was no guarding or rigidity. Examination of other organ systems was normal. Initial laboratory investigations, including hematological and biochemical profiles, were normal. The pregnancy test was negative.

**Figure 1:**
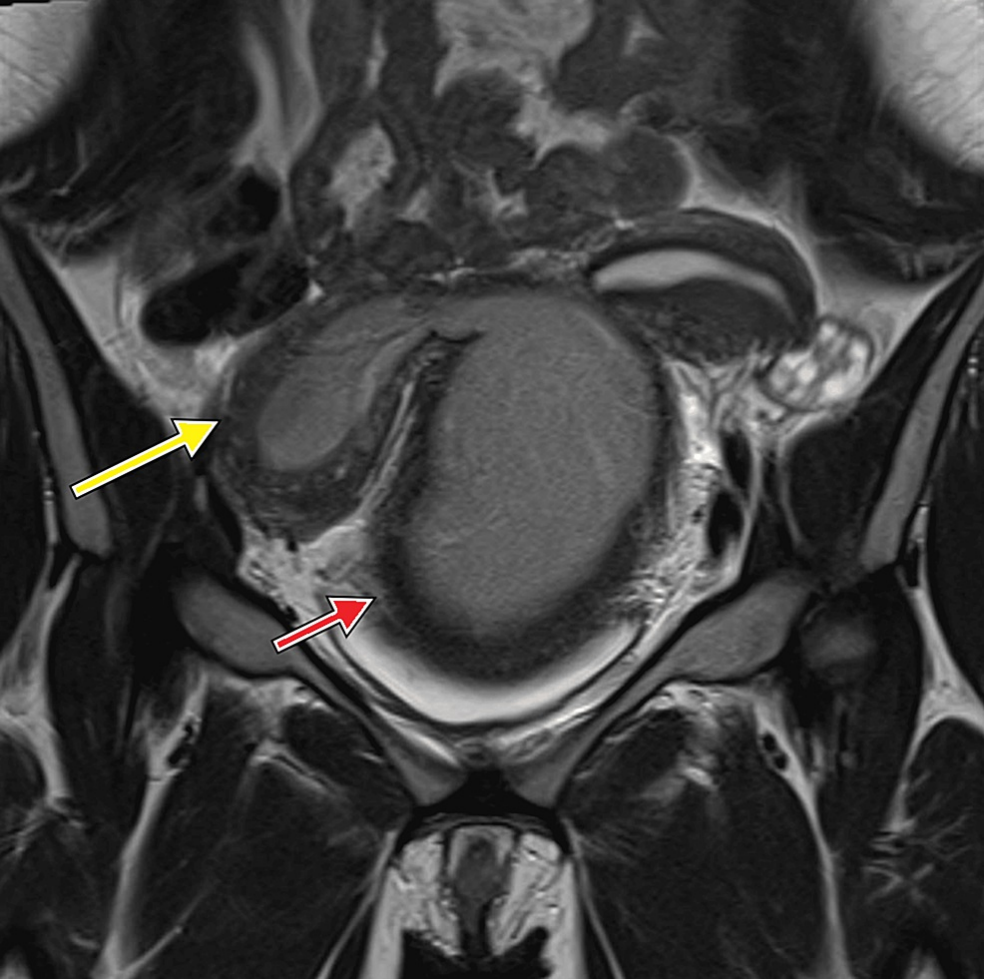
Coronal MR image showing a didelphys uterus with a dilated right endometrial cavity (yellow arrow) and dilated right hemivagina (red arrow). MR: magnetic resonance

**Figure 2:**
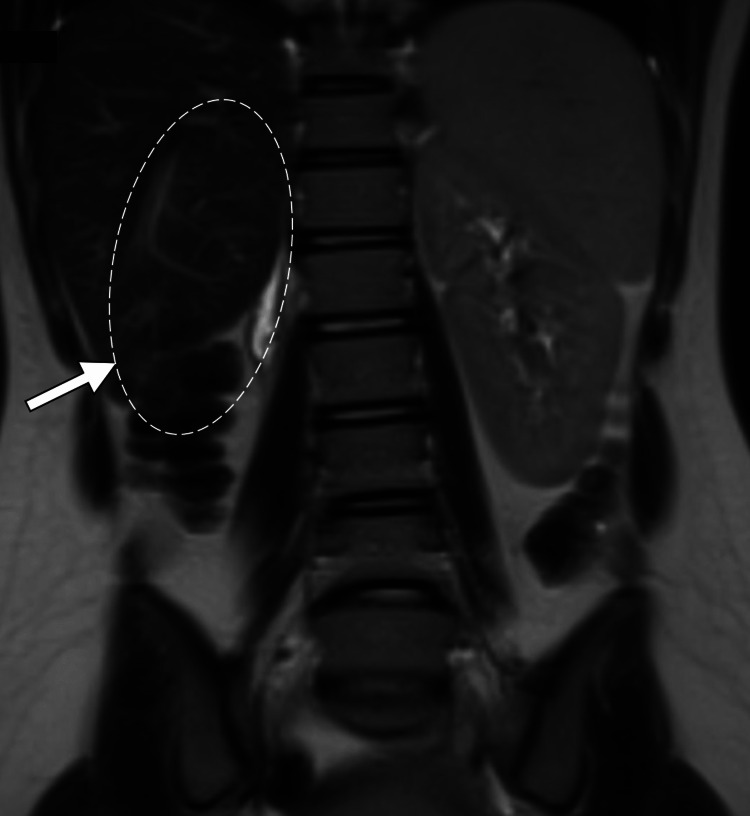
Coronal T2-weighted MR image showing right renal agenesis (arrow). MR: magnetic resonance

An abdominal ultrasound examination was performed to rule out ovarian torsion, but it showed a normal appearance of the ovaries. It also identified a large collection in the rectouterine pouch with unclear uterine morphology. Magnetic resonance imaging was performed, and the duplication of the uterus, cervix, and vagina was identified. The right hemivagina was obstructed, resulting in significant ballooning of the hemivagina, and measured 8.4 cm × 9.0 cm × 12.2 cm (anteroposterior × mediolateral × craniocaudal). The right endometrial cavity was also distended with fluid content. Both the right hemivagina and the right endometrial cavity had a high signal intensity on the T1-weighted images and a low signal intensity on the T2-weighted images, indicating blood content. Such findings were consistent with hematometrocolpos. The left hemivagina and left endometrial cavity were unremarkable. Both ovaries had normal size and morphology. Right renal agenesis with compensatory hypertrophy of the left kidney was observed. The constellation of uterine didelphys, obstructed hemivagina, and ipsilateral renal agenesis represented the diagnosis of Herlyn-Werner-Wunderlich syndrome (Figure 1 and Figure 2).

The diagnosis was discussed with the patient and her parents, and afterward, the patient was referred to undergo the required surgical management. The patient underwent vaginoplasty with resection of the vaginal septum to relieve the obstruction. At follow-up visits, the patient did not have a recurrence of symptoms. She had normal periods after surgery with a resolution of dysmenorrhea.

## Discussion

Herlyn-Werner-Wunderlich syndrome, also known as obstructed hemivagina and ipsilateral renal anomaly syndrome, is a complex type of Müllerian anomaly of rare incidence. The first case of this syndrome was reported in 1922 by Purslow [3]. The association of obstructed hemivagina and ipsilateral renal agenesis was described by Herlyn and Werner in 1977 [3]. Wunderlich described the association of renal agenesis and the bicornuate uterus with the hematocervix [3].

The embryological pathogenesis of Herlyn-Werner-Wunderlich syndrome was not fully understood, as the classic theory of the development of the female reproductive tract does not provide a clear explanation of the complex findings of the Herlyn-Werner-Wunderlich syndrome. The classic theory suggests that the upper part of the vagina develops from the Müllerian duct and the lower part develops from the urogenital sinus. The fusion of these parts forms the vaginal plate, which canalizes later to develop the vaginal lumen [1-3]. Although this theory may explain some Müllerian anomalies, it does not explain the association with renal agenesis in Herlyn-Werner-Wunderlich syndrome.

In 1992, Acein et al. [4] postulated a new theory for the development of the female reproductive tract, providing a better explanation for Herlynq-Werner-Wunderlich syndrome. This theory holds that the uterus and cervix are formed by Müllerian ducts. However, the vagina develops entirely from the Wolffian ducts, which also form the ureteric bud of the kidneys. Wolffian ducts are also essential for the normal positioning of Müllerian ducts. Therefore, a developmental anomaly of one of the Wolffian ducts could lead to the failure of ipsilateral kidney development and duplication of the uterus and cervix.

As in the present case, almost all cases of Herlyn-Werner-Wunderlich syndrome are clinically misdiagnosed initially because of the misleading clinical manifestation and the rare incidence of Herlyn-Werner-Wunderlich syndrome [3]. Patients with Herlyn-Werner-Wunderlich syndrome usually become symptomatic shortly after menarche because of the development of hematometrocolpos [3]. Some patients may get diagnosed in early infancy because of the collection of secretions due to maternal hormones [5].

Magnetic resonance imaging is the most accurate imaging modality of choice for the diagnosis of Herlyn-Werner-Wunderlich syndrome, as it has an excellent evaluation of soft tissues and can provide clear details of the uterine cavity and associated anomalies [2]. Early diagnosis and management are important to resolve obstructive symptoms and prevent the development of endometriosis from retrograde menstruation. The definitive treatment of Herlyn-Werner-Wunderlich syndrome is surgical excision of the vaginal septum and patients can have a normal sexual life [3]. Successful pregnancy in a previously obstructed didelphys uterus in Herlyn-Werner-Wunderlich syndrome has been reported [6].

## Conclusions

Herlyn-Werner-Wunderlich syndrome is a complex type of congenital uterine anomaly. Although rare, Herlyn-Werner-Wunderlich syndrome should be on the differential diagnoses of the presentation of severe dysmenorrhea. The case highlighted an emergency presentation of this syndrome with acute abdominal pain. The diagnosis was readily made by magnetic resonance imaging. Prompt diagnosis of Herlyn-Werner-Wunderlich syndrome is essential as the management is straightforward through vaginoplasty and resection of the septum. This provides quick relief to the patient from her symptoms and prevents complications such as endometriosis.
